# Immune-mediated hematological disease in dogs is associated with alterations of the fecal microbiota: a pilot study

**DOI:** 10.1186/s42523-023-00268-2

**Published:** 2023-09-29

**Authors:** P.-Y. Liu, D. Xia, K. McGonigle, A. B. Carroll, J. Chiango, H. Scavello, R. Martins, S. Mehta, E. Krespan, E. Lunde, D. LeVine, C. L. Fellman, R. Goggs, D. P. Beiting, O. A. Garden

**Affiliations:** 1 Department of Pathobiology and Population Sciences, The Royal Veterinary College, Royal College Street, London, NW1 0TU UK; 2 School of Medicine, College of Medicine, National Sun Yat-sen University, Kaohsiung, 804201 Taiwan; 3 Department of Clinical Sciences and Advanced Medicine, School of Veterinary Medicine, University of Pennsylvania, 3900 Spruce Street, Philadelphia, PA 19104 USA; 4 Department of Pathobiology, School of Veterinary Medicine, University of Pennsylvania, 380 South University Avenue, Philadelphia, 19104 USA; 5 Department of Veterinary Clinical Sciences, College of Veterinary Medicine, Iowa State University, 1809 South Riverside Drive, Ames, IA 50011 USA; 6 Department of Clinical Sciences, College of Veterinary Medicine, Auburn University, 1220 Wire Road, Auburn, AL 36849 USA; 7 Department of Clinical Sciences, Cummings School of Veterinary Medicine, Tufts University, North Grafton, MA 01536 USA; 8 Department of Clinical Sciences, College of Veterinary Medicine, Cornell University, 930 Campus Road, Box 31, Ithaca, NY 14853 USA; 9 Present Address: Dean’s Office, School of Veterinary Medicine, Louisiana State University, Skip Bertman Drive, Baton Rouge, LA 70803 USA

**Keywords:** Hemolytic anemia, Thrombocytopenia, Biomarker, Treponema, *Clostridium septicum*, *Escherichia coli*, Immune-mediated disease, Dog, Microbiome

## Abstract

**Background:**

The dog is the most popular companion animal and is a valuable large animal model for several human diseases. Canine immune-mediated hematological diseases, including immune-mediated hemolytic anemia (IMHA) and immune thrombocytopenia (ITP), share many features in common with autoimmune hematological diseases of humans. The gut microbiome has been linked to systemic illness, but few studies have evaluated its association with immune-mediated hematological disease. To address this knowledge gap, 16S rRNA gene sequencing was used to profile the fecal microbiota of dogs with spontaneous IMHA and ITP at presentation and following successful treatment. In total, 21 affected and 13 healthy control dogs were included in the study.

**Results:**

IMHA/ITP is associated with remodeling of fecal microbiota, marked by decreased relative abundance of the spirochete *Treponema* spp., increased relative abundance of the pathobionts *Clostridium septicum* and *Escherichia coli*, and increased overall microbial diversity. Logistic regression analysis demonstrated that *Treponema* spp. were associated with decreased risk of IMHA/ITP (odds ratio [OR] 0.24–0.34), while Ruminococcaceae UCG-009 and Christensenellaceae R-7 group were associated with increased risk of disease (OR = 6.84 [95% CI 2–32.74] and 8.36 [95% CI 1.85–71.88] respectively).

**Conclusions:**

This study demonstrates an association of immune-mediated hematological diseases in dogs with fecal dysbiosis, and points to specific bacterial genera as biomarkers of disease. Microbes identified as positive or negative risk factors for IMHA/ITP represent an area for future research as potential targets for new diagnostic assays and/or therapeutic applications.

**Supplementary Information:**

The online version contains supplementary material available at 10.1186/s42523-023-00268-2.

## Background

Nearly half of all American households own dogs as companion animals (American Pet Products Association Survey 2015–2016) and dogs are increasingly recognized as a large animal model of human diseases [1–3]. Immune-mediated hematological disease, including immune-mediated hemolytic anemia (IMHA) and immune thrombocytopenia (ITP), is an important cause of morbidity and mortality in dogs. IMHA shares many of the features of warm autoimmune hemolytic anemia in people [4]. Similarly, ITP in the two species shows several common characteristics [5–8]. Erythrocytes or platelets become bound by immunoglobin, leading to opsonization and phagocytosis by macrophages [9–11]. As a result of this process, canine patients present with anemia or thrombocytopenia. First-line therapy involves immunosuppression with corticosteroids, but up to 70% of canine patients with IMHA and up to 20% of patients with ITP succumb to their disease or are euthanized because of aggressive autoimmunity [12–17].

Numerous environmental triggers, including infections, toxins, drugs, parasites, or neoplasia [11, 18–22], have been implicated in the development of autoimmune disease. Notably, there is increasing recognition that the “exposome”—environmental factors that include the patient’s mucosal microbiota—impacts the manifestation of autoimmune disease in humans [23, 24]. Over the past 10 years, a growing evidence base has demonstrated that gut microbiota are crucial for host function and nutrient availability [25–27], xenobiotic detoxification [28–30], and immune system maturation and regulation [31, 32]. Multiple diseases, such as inflammatory bowel disease [33], hepatic steatosis [34], atherosclerosis [35], Parkinson’s disease [36], and colorectal cancer [37], are associated with alterations in the gut microbiome, but molecular evidence of a causal pathogenic role remains unclear [38]. Dogs and humans share similar disease-associated responses in gut microbial composition [39–41], suggesting that inferences made in a canine model are relevant to human disease, with homologous cross-species pathomechanisms [42, 43]. Dysbiosis has been documented in human ITP patients in several studies and is speculated to contribute to pathogenesis [44, 45]. One study of human ITP patients found a link to alterations in the intestinal microbiome and treatment response [46]. Similarly, our preliminary data suggested changes in the intestinal microbiome in dogs with ITP or IMHA, including enrichment of potential pathogens (*Clostridium septicum* and *Escherichia coli*) [47]. A case report describing fecal microbiota transplantation to treat human ITP raises awareness of the therapeutic potential of this strategy, andan ongoing clinical trial is evaluating the efficacy of probiotics in human ITP [48].

In the current study, we hypothesize that alterations in the gut microbiome, inferred from fecal microbiota, are a hallmark of immune-mediated hematological diseases in dogs. Characterizing the fecal microbiota of healthy and diseased dogs, we sought to identify microbial biomarkers of IMHA/ITP in this model species.

## Results

### Study population

**Fig. 1:**
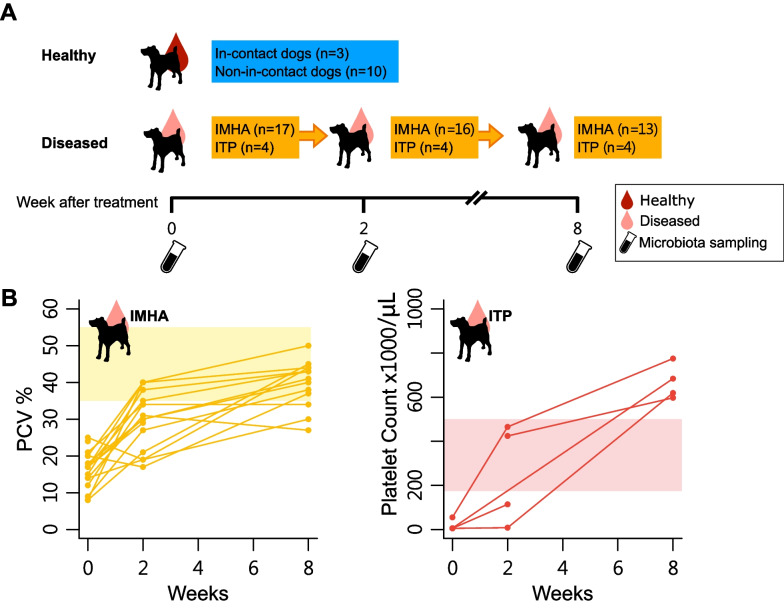
Study design of a survey of gut microbiota in canine immune-mediated hematological disease. **A** Fecal samples of healthy dogs were collected once both from the disease in-contact and non-in-contact dogs. Samples of diseased dogs were collected at presentation (week 0/baseline) and week 2 and week 8 after treatment. **B** Assessments of the clinical response of IMHA (PCV %) or ITP (platelet count × 1000/μL) patients at weeks 0, 2, and 8. The normal values of PCV and platelet counts are depicted by shading. IMHA: immune-mediated hemolytic anemia, ITP: immune thrombocytopenia, PCV: packed cell volume

A total of 31 patients were initially recruited. Twenty-one patients, comprising 17 dogs with IMHA and four dogs with ITP, were responsive to immunosuppressive treatment and were included in the study (Fig. 1A). In addition, 13 healthy dogs were sampled, comprising three ‘in contact’ controls from the same households as affected animals and 10 that were not in contact with affected animals (Fig. 1A). Response to immunosuppressive treatment was defined by an increase in packed cell volume in IMHA, or platelet count in ITP, without relapse, within the first eight weeks of treatment (Fig. 1B). Samples were collected from affected animals at baseline, and two and eight weeks after initiating treatment.

During the study period, 15 of the 21 reported cases were prescribed antimicrobial therapy, including amoxicillin with clavulanic acid (1), ampicillin (1), enrofloxacin (2), doxycycline (4), and metronidazole (10). The beta diversity profile of fecal samples from dogs treated with antimicrobials overlapped with that of dogs not treated with antimicrobials (p = 0.21 by PERMANOVA test; Additional file 1: Figure S1).

### Characterization of fecal microbiota in study dogs reveals consistent mammalian patterns

The qualified dataset contained an average of 49,333 non-chimeric reads, ranging from 27,115 to 71,579 reads/sample (median = 48,690 reads/sample; mean = 49,333 reads/sample) after DADA2 amplicon sequence variant (ASV) de-noising. Broad taxonomic representation of fecal microbiota was similar in healthy and diseased dogs at baseline (Additional file 1: Figure S2). In descending order by relative abundance (> 1% on average), Firmicutes, Bacteroidetes, Spirochaetes, Euryarchaeota and Proteobacteria were the dominant phyla in most of the healthy and baseline disease samples. Firmicutes and Bacteroidetes accounted for over 50% of gut microbiota. *Treponema* (Spirochaetes), *Methanobrevibacter* (Euryarchaeota), and *Escherichia*-*Shigella* (Proteobacteria) were the dominant genera belonging to the most abundant phyla. To distinguish the primary differences between healthy dogs and those with immune-mediated hematological disease, we combined all unaffected dogs to create a single control group, and all affected dogs to create a single disease group, in subsequent analyses.

### Immune-mediated hematological diseases are associated with fecal microbial alterations

**Fig. 2:**
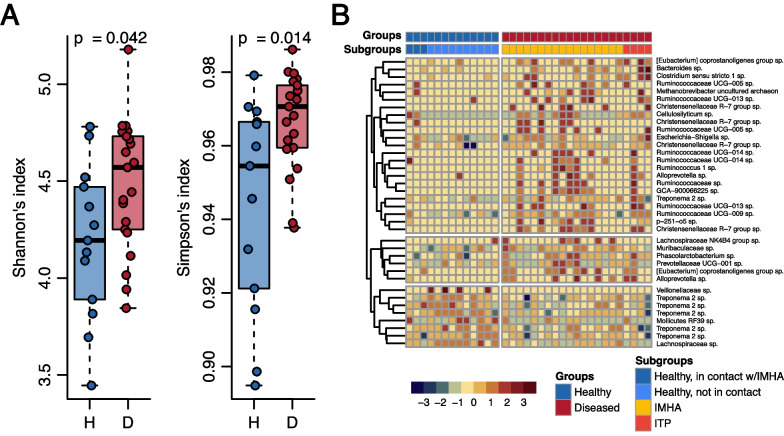
Differential diversity and composition of gut microbiota in healthy and diseased dogs. **A** Alpha diversity measured by the Shannon’s index and Simpson’s index. The diseased dogs had significantly higher Shannon’s and Simpson’s indices. **B** Baseline differential abundance heatmap of gut microbiota between healthy and diseased dogs. Thirty-seven significantly differentiated features (amplicon sequence variants; ASVs) were identified by the DESeq2 test (p < 0.05 and standard error of the log_2_ fold change estimate, lfcSE < 4)

Fecal microbial composition, including abundance and diversity, was analyzed in detail in the healthy and IMHA/ITP dogs to determine the differences between disease status at baseline, prior to initiation of immunosuppressive therapy. Alpha diversity, as measured by Shannon’s index and Simpson’s index, was higher in affected dogs (Fig. 2A).

We used DESeq2 [49] to identify microbes that were differentially abundant between healthy and diseased dogs (*p* < 0.05 and standard error of the log_2 _fold change estimate [lfcSE] < 4) (Fig. 2B). Eight taxa were enriched in the healthy dogs, predominantly belonging to the genus *Treponema*. Twenty-nine taxa were enriched in the affected dogs, including several potential pathogens such as *Clostridium septicum* (the closest NCBI RefSeq annotation for *Clostridium* sensu stricto 1 in Fig. 2B) and *Escherichia coli* (the closest NCBI RefSeq annotation for *Escherichia*-*Shigella* in Fig. 2B).

### Distinct fecal microbes predict risk of immune-mediated hematological disease

**Fig. 3:**
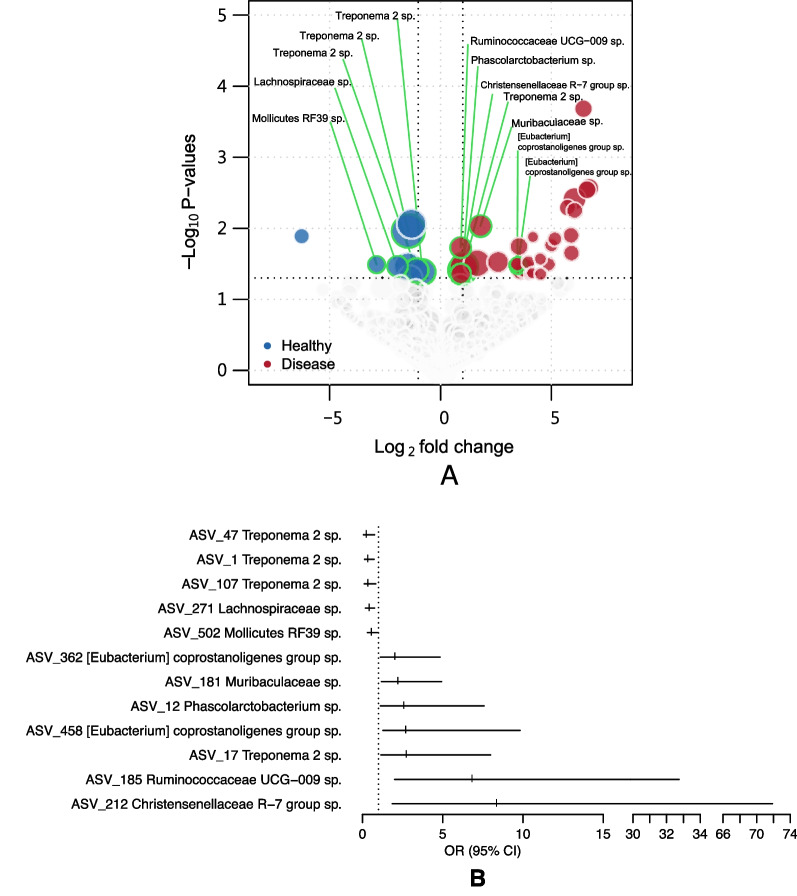
Risk of disease conferred by the differential abundance of microbial amplicon sequence variants (ASVs) identified by odds ratios (OR). **A** Significant ASVs identified by DESeq2 test were tested by logistic regression models. **B** Models passing a p < 0.05 threshold are presented with odds ratios (OR) and 95% confidence intervals

Since we observed several taxa associated with canine IMHA/ITP (Fig. 3A), we used logistic regression analysis to test whether these taxa would predict the risk of developing IMHA/ITP. In total, we observed 12 taxa associated with either increased or decreased odds of developing IMHA/ITP (Fig. 3B and Additional file 1: Table S1). Amongst these were three taxa belonging to the genus *Treponema* associated with decreased risk of IMHA/ITP (odds ratio [OR] = 0.24–0.34; see details in Additional file 1: Table S1). Analysis of the ASVs from these taxa showed that the closest species annotations were *Treponema bryantii* (94.07–98.02% identity) and *Treponema pectinovorum* (94.47% identity). We also identified seven taxa, including one *Treponema* sp. (*Treponema parvum*; 92.10% identity; OR = 2.74, 95% confidence interval [CI] = 1.15–7.98), two *Eubacterium coprostanoligenes* (92.89% identity, OR = 2.03, 95% CI = 1.12–4.83 and 90.51% identity, OR = 2.7, 95% CI = 1.28–9.82) and *Phascolarctobacterium succinatutens* (99.60% identity, OR = 2.58, 95% CI = 1.12–7.57), associated with increased risk of IMHA/ITP, with odds ratios ranging from 2.03 to 8.36 (Additional file 1: Table S1). Two of the taxa most strongly associated with disease were Ruminococcaceae UCG-009 (OR = 6.84, 95% CI = 2–32.74) and Christensenellaceae R-7 group (OR = 8.36, 95% CI = 1.85–71.88), with the closest species annotations being *Papillibacter cinnamivorans* (92.89% identity) and *Novibacillus thermophilus* (88.54% identity), respectively.

### Fecal microbial alterations persist with remission of disease following treatment

**Fig. 4:**
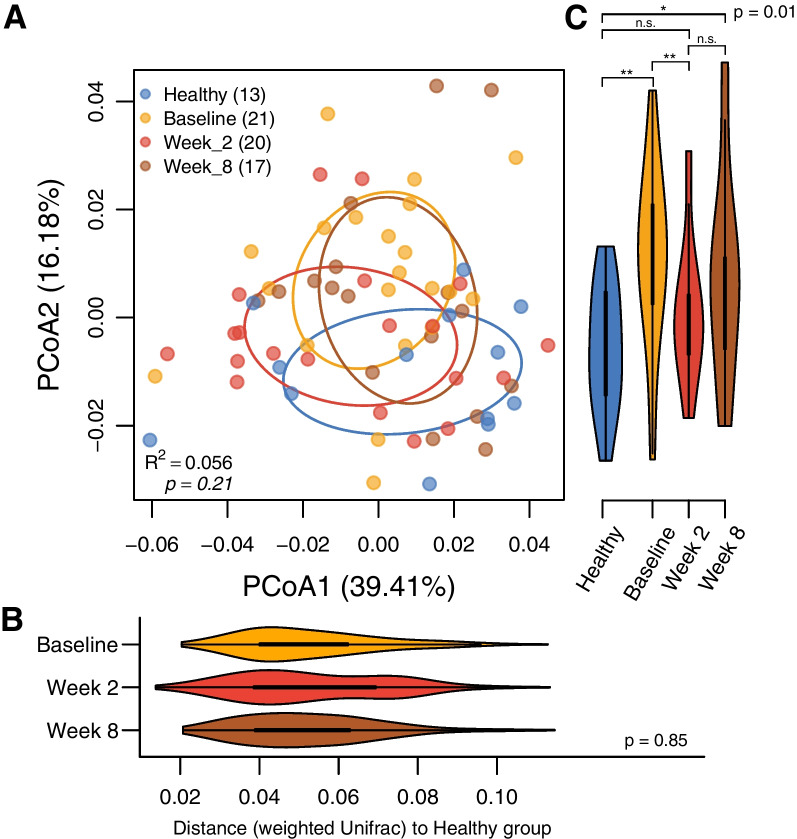
**A** Beta diversity profile (weighted Unifrac distance) of gut microbiota among healthy and diseased dogs at baseline, and weeks 2 and 8. **B** Time course comparison of the diseased group's microbiota measured by distance (weighted Unifrac) to the healthy group. **C** Violin plots demonstrate microbial composition among groups or time points along axis 2 (Kruskal–Wallis test among groups, p = 0.01). The significance of pairwise tests by Dunn's test is labelled as *p < 0.05, **p < 0.01

We next asked whether disease-associated changes in the fecal microbiota of IMHA/ITP dogs resolved with treatment. Treatment with immunosuppressive drugs yielded robust recovery of hematological parameters in the affected dogs included in this study (Fig. 1B). Beta diversity analysis showed no significant shift in microbiome structure following treatment (Fig. 4A, p = 0.21 by PERMANOVA test; Fig. 4B, p = 0.85 by Kruskal–Wallis test). Microbial changes along PC2, accounting for approximately 16% of the total variance in our data set, showed a possible association with treatment, but changes, if any, were subtle (Fig. 4C).

**Fig. 5:**
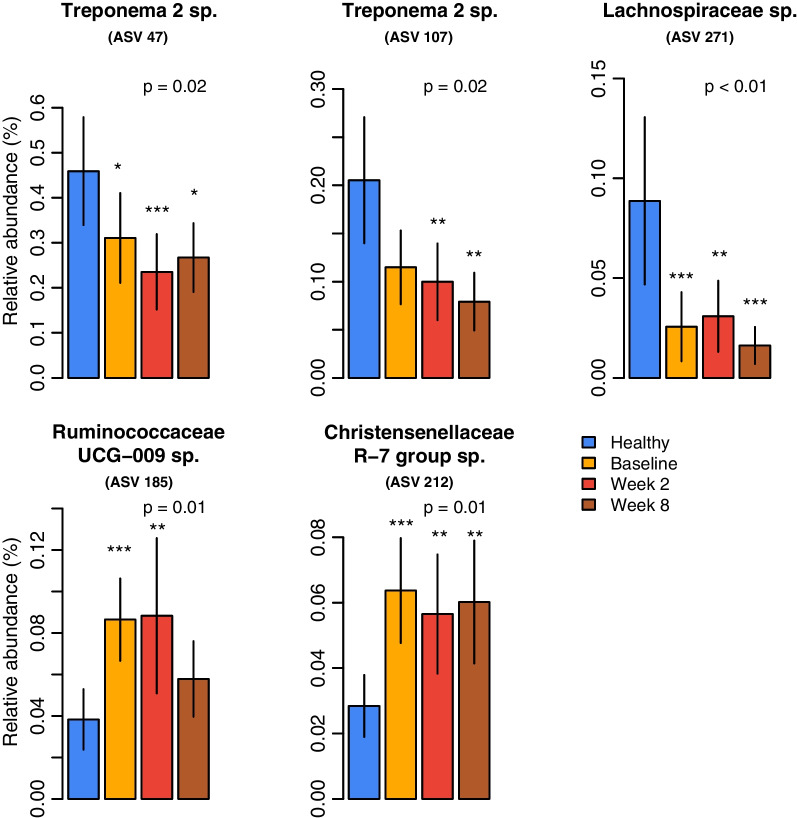
The relative abundance alterations of no-risk versus risk taxa among groups and time points. Five significantly altered taxa were identified by a Kruskal–Wallis test with Dunn’s post-hoc test (compared to the healthy group). The significance of pairwise tests is labelled as *p < 0.05, **p < 0.01, ***p < 0.001

We examined relative abundance of the 12 risk-associated taxa identified from logistic regression analysis of baseline samples (Fig. 3 and Additional file 1: Table S1). Relative abundance of five taxa showed significant differences from the healthy group (Fig. 5). *Treponema* (ASV 47 and ASV 107) and Lachnospiraceae (ASV 271) showed lower relative abundance in diseased dogs throughout treatment. In contrast, Ruminococcaceae UCG-009 (ASV 185) and Christensenellaceae R-7 group (ASV 212) show higher relative abundance in diseased dogs. In addition, the Christensenellaceae R-7 group (ASV 212) remained more abundant throughout treatment, consistent with changes in beta diversity. In all cases, there were no significant changes in relative abundance with treatment.

## Discussion

Our study is the first to examine the fecal microbiota of dogs with the two most common immune-mediated hematological diseases in this species. Although we regard this as a pilot study, the differential abundance of several bacterial species within the diseased dogs was notable. These differences raise the intriguing possibility that fecal microbial composition may play a protective or pathogenic role in immune-mediated diseases of the blood. To our surprise, there were no significant changes in fecal microbial populations with treatment over the eight-week period of observation despite remission of disease, arguing against rapid shifts in the fecal microbiome that mirror those of clinical disease.

Several species of the genus *Treponema* showed lower abundance in the diseased dogs, prompting us to speculate that they may play a protective role in health. In contrast, Ruminococcaceae and Christensenellaceae R-7 group species showed higher abundance in the diseased dogs, raising the possibility that they may play a pathogenic role in disease. Several studies in other species have demonstrated the importance of the intestinal microbiome in the initiation and progression of autoimmune disease, driven by mechanisms including the translocation of pathobionts that elaborate proinflammatory molecules, bacterial mimicry of autoantigens, and parallel dysregulation of the metabolome with attenuation of anti-inflammatory pathways [50–53].

Increasing recognition of the role of the intestinal microbiome in distal homeostatic and pathogenic immune pathways suggests that manipulation of the mucosal microbiota with antibiotics, prebiotics, probiotics, synbiotics, or fecal transplantation may represent a novel therapeutic opportunity in autoimmunity [53–56]. Various studies have documented intestinal dysbiosis in human ITP, as well as bacterial signatures that correlate with clinical indices, but the discordance of findings and relative paucity of data underscore the complexity of this field and the need for additional research [44, 45, 57, 58]. Moreover, to the best of our knowledge similar studies have not been performed in autoimmune hemolytic anemia in human patients, which represents an unmet need in this area of medicine [4, 56].

The lower abundance of *Treponema* spp. in diseased dogs in the current study is a novel finding. Although this spirochete genus has been associated with syphilis [59], gastritis [60], Alzheimer’s disease [61], periodontitis and gingivitis [62] in people, and periodontitis in dogs [63–65], the potentially protective role of certain *Treponema* spp. has been poorly characterized. Nguyen and colleagues documented decreased abundance of four core genera, including *Treponema*, in the feces and cecal mucus of mice with complete Freund adjuvant-induced arthritis, a model of rheumatoid arthritis [66]. The synthesis of anti-inflammatory short chain fatty acids by *Treponema* spp. was suggested as a possible protective mechanism, but both their impact on disease and any mechanistic basis of such interactions remain speculative at this juncture. Moreover, one species, *Treponema parvum*, was among those seven taxa associated with increased risk of IMHA/ITP in our study, emphasizing the danger of broad, genus-level statements that ignore species-level nuances.

The increased abundance of Ruminococcaceae and Christensenellaceae R-7 group species in the diseased dogs also represents a novel, albeit unexpected, finding. In general, the Ruminococcaceae are anti-inflammatory, short-chain fatty acid-synthesizing bacteria present in high abundance in the intestinal microbiota of healthy people [67] and dogs [42, 68], and low abundance in human patients with various autoimmune diseases [69, 70], including ITP [58]. Of note in the canine intestinal microbiome is the species *Faecalibacterium prausnitzii*, a strict anaerobe of the phylum Firmicutes, class Clostridia, and order Clostridiales that is considered a hallmark of intestinal microbial health [42, 71]. Nevertheless, individual species within this family may have pathogenic potential. The closest species annotation for Ruminococcaceae UCG-009 is *Papillibacter cinnamivorans*, a bacterium whose abundance is associated with both beneficial outcomes, for example in dogs fed a Mulberry leaf supplement in the treatment of obesity [72], and deleterious outcomes, for example in patients with Parkinson’s disease [73]. Similarly, another species belonging to this family, *Ruminococcus anavus*, was present in greater abundance in the feces of patients with ITP, in which alterations of microbial species correlated with clinical indices [46]. In similar fashion to the Ruminococcaceae, the Christensenellaceae are generally considered to be anti-inflammatory healthy bacteria [74], present in lower abundance in states of autoimmunity [75]. However, little is known of the species we identified, *Novibacillus thermophilus*, and the possibility remains that intestinal microbial perturbations represent protective rather than pathogenic mechanisms, triggered as a response to immune-mediated disease [76].

Other bacteria positively associated with disease in our study included *Clostridium septicum*, *Eubacterium coprostanoligenes*, and *Phascolarctobacterium succinatutens*. While none of these bacteria has been associated with immune-mediated disorders, they have been associated with other disease entities. Documented in the fecal microbiota of healthy people and dogs, *Clostridium septicum* has been associated with sepsis [77], type 3c diabetes mellitus [78], and colorectal cancer [79–82]. Bacteria within the genus *Eubacterium* are generally considered beneficial to health, producing butyrate and metabolizing bile acids and cholesterol [83, 84]. *Eubacterium coprostanoligenes* is a cholesterol-reducing anaerobic coccobacillus that has been implicated in the phenomenon of manure “foaming” [85], but little is known about its pathogenic potential. Its abundance in the feces of children with autism spectrum disorder was positively correlated with gastrointestinal symptoms [86]. Fecal microbiota transplantation decreased the abundance of this bacterium, with an improvement in both behavioral and gastrointestinal symptoms, suggesting a possible association with neurobehavioral disease [86]. Its potential role in autoimmune disease remains unknown. The abundance of bacteria within the genus *Phascolarctobacterium* has been associated with neuropsychiatric disorders [87], psoriasis [88], Hashimoto thyroiditis [89], and diabetes mellitus [90, 91], suggesting pathogenic potential in a variety of settings. There was a positive correlation between the concentration of soluble interleukin-2 receptor in psoriasis patients and fecal abundance of *Phascolarctobacterium *[88], raising the possibility that this bacterium may contribute to the pro-inflammatory phenotype of this disease. *Phascolarctobacterium succinatutens* has been implicated as a signature species in human metabolic dysfunction fatty liver disease [92], although its abundance was reduced in the intestinal microbiome of obese cats [93]. This dichotomy once again highlights the challenges inherent in associating specific bacteria with specific diseases within and across mammalian taxa.

The lack of significant changes in the fecal microbiota with treatment of the IMHA/ITP patients in this study, all of which responded to immunosuppression within eight weeks, was unanticipated. We had speculated that dysbiotic signatures associated with disease would normalize in parallel with clinical remission, as has been found in several diseases of immune-mediated etiology in human patients and rodent models, including rheumatoid arthritis [66, 94], uveitis [95], keratoconjunctivitis sicca [51], neuropsychiatric disorders [86], diabetes mellitus [96], and autoimmune thyroid disorders [97]. It is possible the changes we documented in fecal microbiota may take longer than eight weeks to normalize, or that the apparent dysbiosis in these patients may never completely resolve while they are being treated. Furthermore, several dogs received antimicrobial drugs during the time immunosuppressive therapy was administered, potentially inhibiting normalization of fecal microbial communities despite the apparent absence of an antimicrobial impact on beta diversity (Additional file 1: Figure S1). There is also increasing recognition that glucocorticoids negatively impact intestinal microbial diversity in several species [98–102]. Nevertheless, changes in the microbiota induced by glucocorticoids are thought to underlie their beneficial impact in systemic lupus erythematosus [103, 104] and inflammatory bowel disease [105], underscoring the complexity of the inter-relationship between intestinal microbial composition, disease status, and the influence of therapeutic drugs. If dysbiosis is a primary pathogenic driver of IMHA and ITP, we hypothesize that delayed normalization of microbial composition could contribute to the tendency of patients to relapse with premature cessation of immunosuppressive treatment.

There were several shortcomings of this study, from which only preliminary conclusions can therefore be drawn. A modest number of dogs with IMHA and ITP were recruited from multiple centers of wide geographical dispersion, managed by different clinicians with different clinical approaches. Given the limited number of cases, both diseases were considered as one group, potentially undermining our ability to discern disease-specific signatures. We included only samples from dogs responding to immunosuppressive treatment, precluding the assessment of bacterial signatures associated with unresponsive, or relapsing, disease. Most of the healthy control dogs were from different households from the cases recruited into the study, giving us no opportunity to control for differences attributable to specific environmental exposure. Patients were tracked for only eight weeks, with the risk of missing longer-term changes in the fecal microbiota. There were also limitations in the taxonomic resolution of short-reading sequences. Nevertheless, several notable observations were made, robust to the confounding influence of recruiting center, specific disease, and other variables. Pilot data were generated that motivate further studies in a larger number of patients in the future.

In conclusion, immune-mediated hematological disease in dogs was associated with alterations in fecal microbiota in this small cohort of patients. Whether these changes were of primary pathogenic potential or epiphenomena remains unknown. Novel treatments that aim to restore healthy microbial composition may provide an adjunct to current immunosuppressive approaches in canine IMHA and ITP. Further research at the nexus of the intestinal microbiome and autoimmune disease in this species is warranted.

## Methods

### Inclusion criteria, treatment regimen, and sample collection

Immune-mediated hemolytic anemia was diagnosed by documenting a packed cell volume (PCV) of < 35% or a hemoglobin concentration of < 11 g/dL, associated with two examples of antibodies directed against erythrocyte antigens, as indicated by either a positive saline agglutination or Coombs’ test result, or moderate to marked spherocytosis [106]. When only one of the above tests suggestive of antibodies was available, the additional indicator of hemolysis (i.e. hyperbilirubinemia, hemoglobinuria, hemoglobinemia, erythrocyte ghosts) fulfilled inclusion criteria. Idiopathic (non-associative) disease was diagnosed using a standardized diagnostic approach ruling out potential trigger factors—including iatrogenic causes, neoplasia, and infection—as assessed by history, physical examination, imaging of the thorax and abdomen, and screening for vector-borne pathogens [106].

Immune thrombocytopenia was diagnosed by documenting a platelet count of less than 50,000/µL in a dog with no macroplatelets (confirmed on a blood smear) and no evidence of associative disease or disseminated intravascular coagulation, as assessed by imaging of the thorax and abdomen, screening for vector-borne pathogens, and coagulation tests. A canine bleeding assessment tool (DOGiBAT) score of at least 2 was required for inclusion [107].

In both IMHA and ITP, treatment-naïve cases were recruited, defined by the administration of no more than three doses of an immunosuppressive drug or biologic in the 28 days preceding presentation, either consecutively or non-consecutively. To minimize confounding factors, cases were excluded if they had received any antimicrobial drugs or pre/probiotics in the five days preceding presentation, or one or more doses of a vaccine or a toxin in the 28 days preceding presentation (Additional file 1: Table S2). Dogs were not allowed to receive antimicrobials during the study period except doxycycline for prophylaxis against vector-borne pathogens (pending test results), metronidazole for non-limiting diarrhea associated with mycophenolate mofetil administration, or drugs to treat secondary infections that arose once the dogs had been recruited (e.g. urinary tract infection) (Additional file 1: Figures S3 and S4). Neither the patients nor control dogs had received long-term antimicrobial drugs prior to recruitment.

All recruited cases received immunosuppressive therapy, comprising initial dexamethasone (0.2–0.3 mg/kg IV q24h; maximum dose 8 mg), followed by prednisone or prednisolone (1.6-3 mg/kg PO q24h; maximum dose 60 mg) and, in most cases, a second immunosuppressive drug (Additional file 1: Figures S3 and S4; Table S2). The administration of blood products was permitted as clinically indicated. After initial recruitment, cases were excluded if clinically significant comorbidities (e.g. diabetes mellitus) were diagnosed. Cases that failed to respond to treatment or that relapsed during the eight-week study period were also excluded from the final dataset. Corticosteroid therapy was tapered gradually, according to a standard protocol [108]. Remission of disease was defined by the attainment of a PCV > 37% (IMHA) or a platelet count of  > 150,000/µL (ITP) and the absence of immune markers of disease. Relapse, if it occurred, was defined by a drop in PCV of at least 5% (IMHA) or a drop in platelet count of at least 50,000/µL (ITP) since the preceding visit.

Patients were recruited from four centers, including the Matthew J Ryan Hospital, University of Pennsylvania (Penn Vet; n = 16), Cornell University Hospital for Animals (n = 12), the Lloyd Veterinary Medical Center, Iowa State University (n = 1), and the Foster Hospital for Small Animals, Tufts University (n = 2). At each time point, a fresh fecal sample was collected by rectal palpation or from a clean surface within 15 min of defecation. Fresh fecal samples were also collected once from healthy control dogs, both from the same household as patients (when available; in-contact, n = 3) and different households (non‒in-contact; n = 10). Inclusion criteria for healthy control dogs included the presence of systemic health based on a history and physical examination; the absence of antimicrobial or pre/pro-biotic consumption within five days of presentation; and the consumption of no more than two doses, either consecutively or non-consecutively, of any drug, including immunosuppressives and biologicals but excluding nutraceuticals, within 28 days of presentation. Neither the patients nor control dogs had consumed raw diets or treats within 28 days of presentation.

Fresh fecal samples of at least 10 g in weight were collected within 15 min of defecation or by rectal palpation, delivered to Penn Vet within three hours of collection, and kept at 4 °C for no more than 48 h, before being treated and stored at − 20 °C. If drop-off at Penn Vet was not possible within three hours of defecation, fecal samples were stored in a − 20 °C freezer (within three hours of defecation) and transported to Penn Vet on ice within 10 days of being placed in the − 20 °C freezer.

### 16S rRNA gene sequencing and data analysis

Bacterial genomic DNA was extracted from homogenized fecal samples using the PowerSoil DNA Isolation Kit (MO BIO Laboratories, Carlsbad, CA) following the manufacturer’s instructions. A mock community genomic DNA library was amplified and sequenced as a quality control sample. Additional controls included extraction of blank-processed samples (water only) to determine background microbial signal. A Nextera dual-index amplicon library construction method targeted the V4 region of the 16S rRNA gene by PCR amplification [109]. Pico-green-based amplicons were sequenced on a MiSeq platform (Illumina, San Diego, CA) using 250 bp paired-end chemistry.

A total of 3,990,059 paired-end sequences were generated for the 71 samples (excluding samples with fewer than 5,000 read counts, blank and mock control samples) from the Illumina MiSeq platform. The 16S rRNA gene amplicon sequences were processed using the Quantitative Insights Into Microbial Ecology 2 (QIIME 2) pipeline (version 2019.10) [110]. Reads were truncated at 220 bp for forward reads and 200 bp for reverse reads, then de-noised using the DADA2 algorithm [111, 112]. Amplicon sequence variants (ASVs) were obtained via the de-noising process with quality filtering and removal of chimeras. Consensus taxonomy was assigned using the classifier-consensus-vsearch plugin (the VSEARCH algorithm) [113, 114] against SILVA NR132 99% 16S rRNA gene sequences [115, 116]. The NCBI 16S RefSeq Nucleotide sequence records (retrieved from https://www.ncbi.nlm.nih.gov/refseq/targetedloci/16S_process/ on January 8th 2022) were downloaded and trained as a BLAST database; the closet taxonomy of each ASV was assigned by using ‘blastn’ algorithm under the criteria of e-value ≤ 1e-5, max_target_seqs = 1, and max_hsps = 1. Representative sequences were aligned with MAFFT v7, and variable positions were then masked. A phylogenetic tree was built with the FastTree 2.1 and then rooted with midpoint.

Microbiome diversity and composition were analyzed in the context of disease status, and then visualized using the MARco [117], vegan [118], and pheatmap [119] packages in R software (version 4.1.2) [120]. A Kruskal–Wallis test and Dunn’s post-hoc test were used for all statistical analyses of group comparisons with a significance level of α = 0.05, and the p values were adjusted with a false discovery rate (FDR). A DESeq2 [49] analysis allowed group comparisons of each feature under the criteria of *p* < 0.05 and lfcSE < 4. Alpha diversity indices were estimated by richness, Shannon’s index, and Simpson’s index. Beta diversity of microbial communities was measured by weighted Unifrac distance [121, 122] using a principal coordinates analysis (PCoA). Heterogeneity was examined using ADONIS tests.

### Supplementary Information

**Additional file 1. Figure S1**. Comparison of the diversity of gut microbiota between dogs receiving antimicrobials (22 samples from 15 diseased dogs collected at baseline, week 2, and week 8) and dogs not receiving antimicrobials (36 samples without record of antimicrobial administration during the study period). No statistically significant difference between the two groups was observed (p = 0.21 by PERMANOVA). Numbers on the symbols represent treatment duration (days). **Figure S2** Baseline dogs’ gut microbiota profile at (A) phylum, (B) class, and (C) genus levels among healthy in-contact controls (Healthy, C), healthy non-in-contact controls (Healthy, NC), and IMHA and ITP dogs. IMHA: immune-mediated hemolytic anemia, ITP: immune thrombocytopenia. **Figure S3** Treatment flow chart for IMHA. **Figure S4** Treatment flow chart for ITP. **Table S1** Immune-mediated hematological disease odds ratios of differentially abundant taxa between healthy and diseased dogs. **Table S2** Inclusion/exclusion criteria applied in the study.

## Data Availability

Raw 16S rRNA gene sequences and metagenomic sequences for all samples used in this study have been deposited in the Sequence Read Archive (SRA) (accession number pending).
