# Supplementary material for: Immune-mediated hematological disease in dogs is associated with alterations of the fecal microbiota: a pilot study
Source: Anim Microbiome. 2023 Sep 29;5:46. doi: 10.1186/s42523-023-00268-2 (PMC10540429; doi:10.1186/s42523-023-00268-2)
Supplement: Supplementary file 1 — Additional file 1. Figure S1. Comparison of the diversity of gut microbiota between dogs receiving antimicrobials (22 samples from 15 diseased dogs collected at baseline, week 2, and week 8) and dogs not receiving antimicrobials (36 samples without record of antimicrobial administration during the study period). No statistically significant difference between the two groups was observed (p = 0.21 by PERMANOVA). Numbers on the symbols represent treatment duration (days). Figure S2 Baseline dogs’ gut microbiota profile at (A) phylum, (B) class, and (C) genus levels among healthy in-contact controls (Healthy, C), healthy non-in-contact controls (Healthy, NC), and IMHA and ITP dogs. IMHA: immune-mediated hemolytic anemia, ITP: immune thrombocytopenia. Figure S3 Treatment flow chart for IMHA. Figure S4 Treatment flow chart for ITP. Table S1 Immune-mediated hematological disease odds ratios of differentially abundant taxa between healthy and diseased dogs. Table S2 Inclusion/exclusion criteria applied in the study. [file 42523_2023_268_MOESM1_ESM.pdf]

Supplementary figures

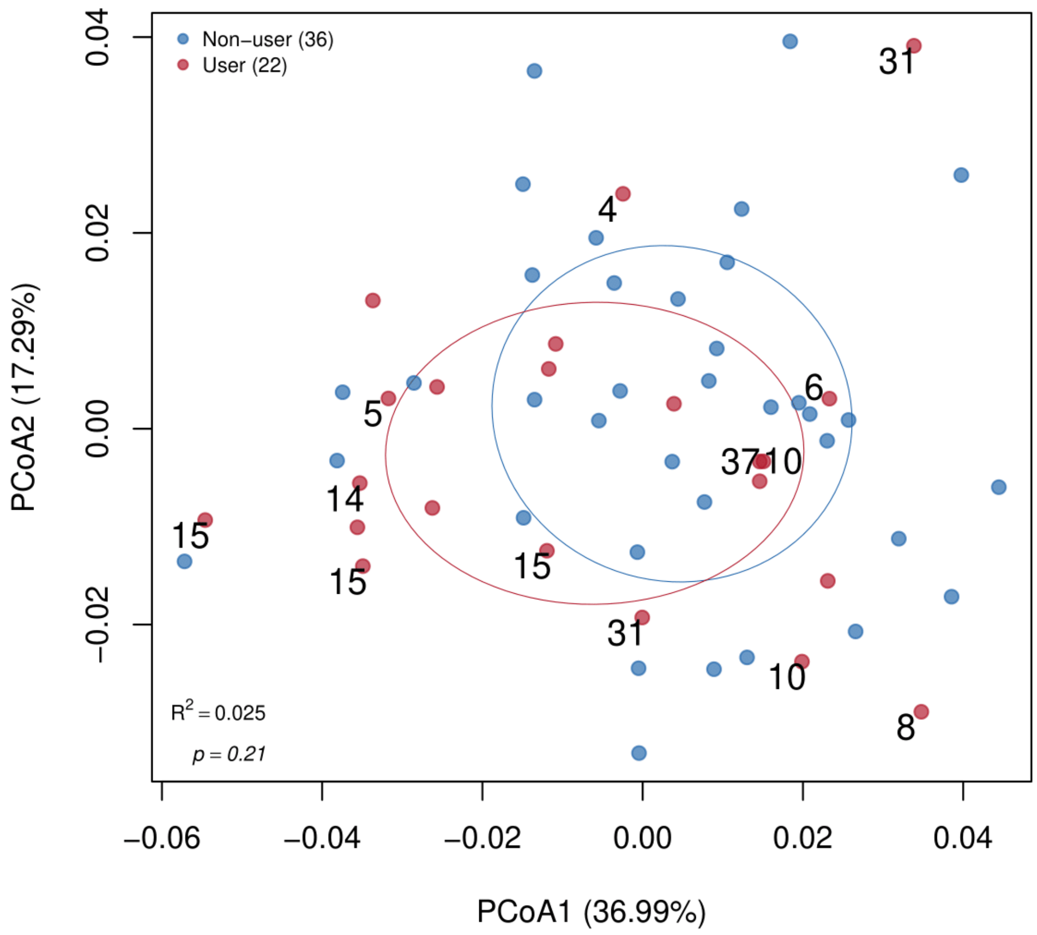


**Figure S1** Comparison of the diversity of gut microbiota between dogs receiving antimicrobials (22 samples from 15 diseased dogs collected at baseline, week 2, and week 8) and dogs not receiving antimicrobials (36 samples without record of antimicrobial administration during the study period). No statistically significant difference between the two groups was observed (p=0.21 by PERMANOVA). Numbers on the symbols represent treatment duration (days).

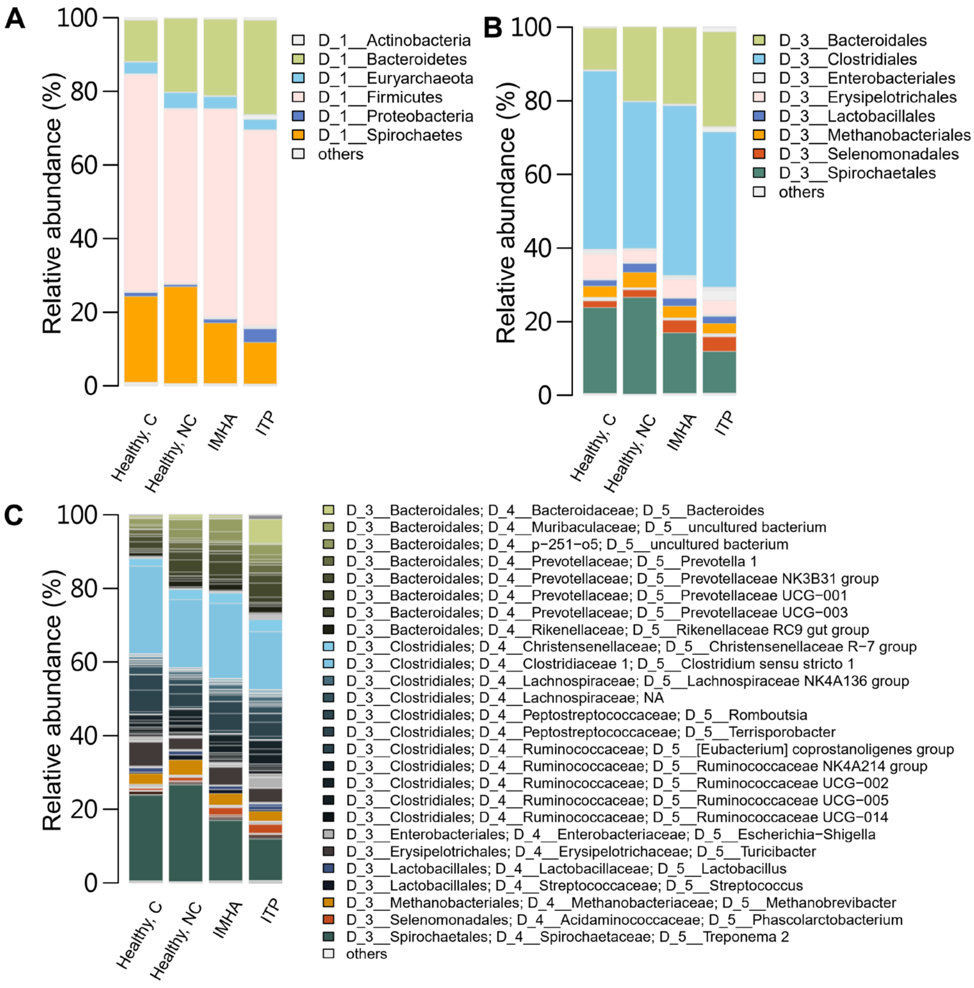


**Figure S2** Baseline dogs' gut microbiota profile at (A) phylum, (B) class, and (C) genus levels among healthy in-contact controls (Healthy, C), healthy non–in-contact controls (Healthy, NC), and IMHA and ITP dogs. IMHA: immune-mediated hemolytic anemia, ITP: immune thrombocytopenia.

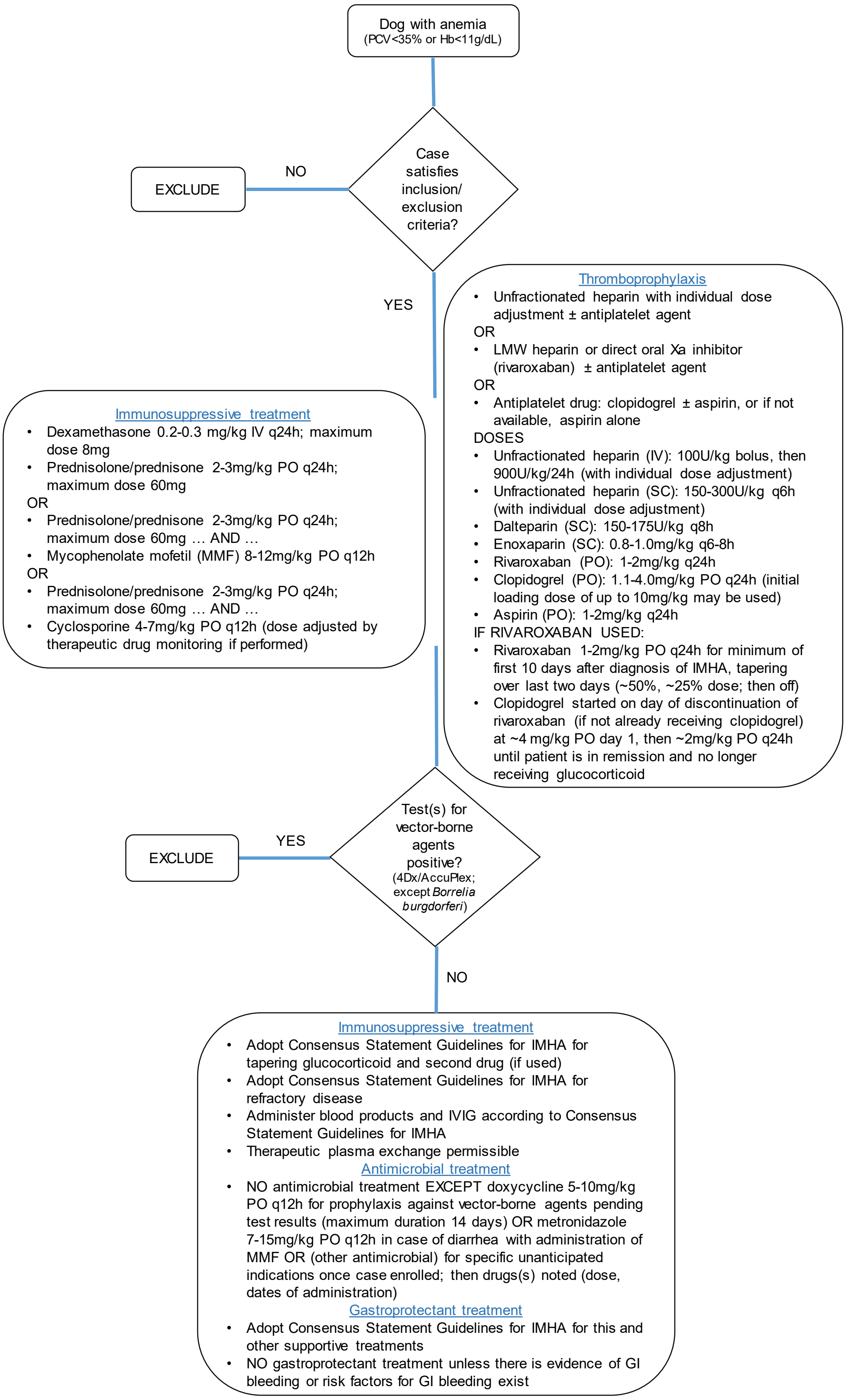


**Figure S3** Treatment flow chart for IMHA.

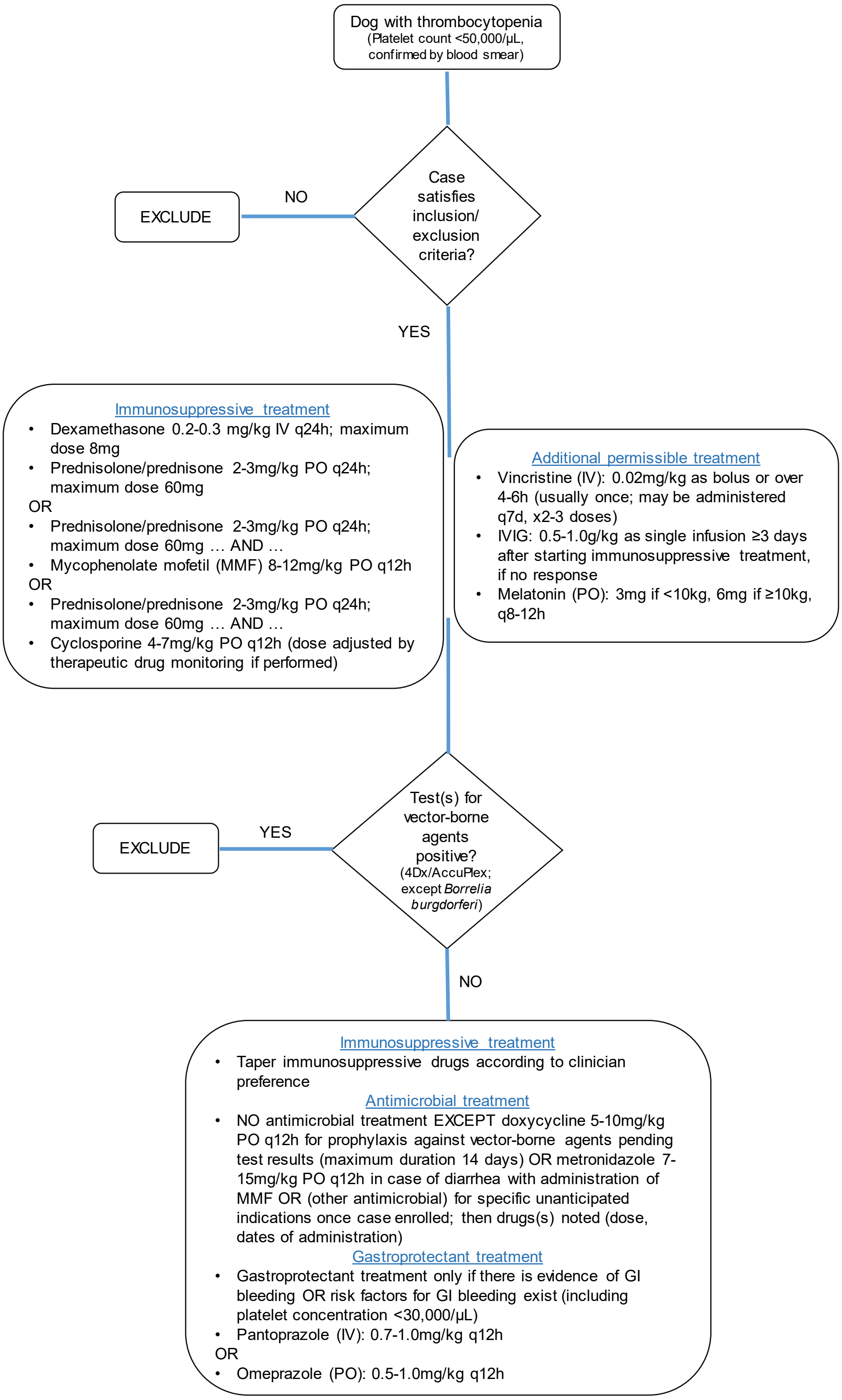


**Figure S4** Treatment flow chart for ITP.

Supplementary tables

**Supplementary Table 1** Immune-mediated hematological disease odds ratios of differentially abundant taxa between healthy and diseased dogs.

| # ASV | SILVA Taxonomy | NCBI Taxonomy (identity %) | Estimate | P-value | OR (95% CI) |
|---|---|---|---|---|---|
| ASV 47 | *Treponema* 2 sp. | *Treponema bryantii* (94.071%) | -1.41 | 0.03 | 0.24 (0.05-0.75) |
| ASV 1 | *Treponema* 2 sp. | *Treponema bryantii* (98.024%) | -1.08 | 0.01 | 0.34 (0.13-0.72) |
| ASV 107 | *Treponema* 2 sp. | *Treponema pectinovorum* (94.466%) | -1.07 | 0.04 | 0.34 (0.1-0.82) |
| ASV 271 | *Lachnospiraceae* sp. | [*Eubacterium*] *rectale* ATCC 33656 (95.257%) | -0.86 | 0.01 | 0.42 (0.18-0.75) |
| ASV 502 | *Mollicutes* RF39 sp. | *Paenibacillus aestuarii* (83.73%) | -0.57 | 0.04 | 0.56 (0.31-0.93) |
| ASV 362 | [*Eubacterium*] *coprostanoligenes* group sp. | *Eubacterium coprostanoligenes* (92.885%) | 0.71 | 0.04 | 2.03 (1.12-4.83) |
| ASV 181 | *Muribaculaceae* sp. | *Muribaculum intestinale* (86.905%) | 0.79 | 0.03 | 2.21 (1.19-4.92) |
| ASV 12 | *Phascolarctobacterium* sp. | *Phascolarctobacterium succinatutens* (99.603%) | 0.95 | 0.045 | 2.58 (1.12-7.57) |
| ASV 458 | [*Eubacterium*] *coprostanoligenes* group sp. | *Eubacterium coprostanoligenes* (90.514%) | 0.99 | 0.03 | 2.7 (1.28-9.82) |
| ASV 17 | *Treponema* 2 sp. | *Treponema parvum* (92.095%) | 1.01 | 0.04 | 2.74 (1.15-7.98) |
| ASV 185 | *Ruminococcaceae* UCG-009 sp. | *Papillibacter cinnamivorans* (92.885%) | 1.92 | 0.01 | 6.84 (2-32.74) |
| ASV 212 | *Christensenellaceae* R-7 group sp. | *Novibacillus thermophilus* (88.538%) | 2.12 | 0.02 | 8.36 (1.85-71.88) |

**Supplementary Table 2** Inclusion/exclusion criteria applied in the study.

| **Criterion** | **Inclusion** | **Notes** |
|---|---|---|
| Diagnosis | IMHA following Consensus Statement guidelines – supportive and diagnostic only<br>OR<br>ITP documented by blood smear confirmation and rule-out of other causes of thrombocytopenia | |
| | Idiopathic disease | FROM 0 TO 28 DAYS PRIOR TO PRESENTATION (i.e. DAYS 0 TO -28 INCLUSIVE)<br>• Has received no more than 2 doses, either consecutively or non-consecutively, of any drug, including immunosuppressives and biologicals (e.g. lokivetmab [Cytopoint®]), but excluding nutraceuticals<br>• Has not been exposed to toxins or received any vaccine dose(s)<br><br>FROM 0 TO 5 DAYS PRIOR TO PRESENTATION (i.e. DAYS 0 TO -5 INCLUSIVE)<br>• Has not received any antimicrobials or pre/pro-biotics<br>AT TIME OF PRESENTATION<br>• No evidence of neoplasia on thoracic and abdominal imaging<br>• No evidence of bacterial or vector-borne infection, but see below for *Borrelia burgdorferi*<br>• Minimum SNAP4Dx (or equivalent) testing for vector-borne agents; *Borrelia burgdorferi* results interpreted according to location. All cases yielding positive immunochromatographic screening test results for *Anaplasma spp.* excluded. |

| | | |
|---|---|---|
| Antimicrobials | Has not received any antimicrobials or pre/pro-biotics within the 5 days prior to presentation (including doxycycline and/or metronidazole) | |
| Immunosuppressive drugs | Has not received more than 2 doses of an immunosuppressive drug, consecutively or non-consecutively, within the 28 days prior to presentation | Immunosuppressive drug(s) include:<br>• Dexamethasone<br>• Prednisone/prednisolone<br>• Azathioprine, mycophenolate mofetil, cyclosporine, leflunomide |
| Informed consent | Informed consent granted | Require signed Informed Consent Form for records |
